# MicroRNA-137 inhibits BMP7 to enhance the epithelial-mesenchymal transition of breast cancer cells

**DOI:** 10.18632/oncotarget.15442

**Published:** 2017-02-17

**Authors:** Xuexiang Ying, Yunpo Sun, Pingqing He

**Affiliations:** ^1^ Department of General Surgery, Shanghai Jiaotong University Affiliated Sixth People's Hospital, 200233, China

**Keywords:** breast cancer (BC), epithelial-mesenchymal transition (EMT), miR-137, bone morphogenetic protein-7 (BMP7)

## Abstract

Bone morphogenetic protein-7 (BMP7) is known to antagonize transforming growth factor β 1 (TGFβ1)-mediated fibrosis through suppressing epithelial-mesenchymal transition (EMT). We recently reported that BMP7 also antagonizes the effects of TGFβ1 in breast cancer (BC) tumorigenesis-related EMT. Nevertheless, the control of BMP7 expression in BC remains ill-defined. Here, we detected significantly lower levels of BMP7 and significantly higher levels of microRNA-137 (miR-137) in the BC specimens, relative to paired adjacent non-tumor breast tissue. BMP7 and miR-137 levels were correlated inversely. Additionally, the high miR-137 levels in BC specimens were correlated with reduced patient survival. *In vitro*, overexpression of miR-137 significantly increased cell EMT and invasion, while depletion of miR-137 significantly decreased cell EMT and invasion in BC cells. The increases in BC cell invasiveness by miR-137 appeared to result from its suppression of BMP7, through direct binding of miR-137 to the 3'-UTR of BMP7 mRNA, thereby blocking its protein translation in BC cells. This study sheds light on miR-137 as a crucial factor that enhances BC cell EMT and invasiveness, and points to miR-137 as a promising innovative therapeutic target for BC treatment.

## INTRODUCTION

Breast cancer (BC) is a common malignant tumor in women worldwide [1]. The transforming growth factor β (TGFβ) superfamily receptor signaling pathway plays a key role in the tumorigenesis of BC [2–5], in which action of TGFβ receptor signaling by its ligand TGFβ1 promotes a biological process called Epithelial-Mesenchymal Transition, which cancer cells use to favor an invasive and metastatic phenotype (EMT) [6–9]. In this process, cancer cells begin to secrete proteinases in order to traverse collagenous extracellular matrix proteins. Bone morphogenetic protein-7 (BMP7) is a well-described matrix proteinase that breaks down collagen type IV, a constituent of the basement membrane. Down-regulation of BMP7 facilitates the metastatic spread of BC cells [10–15]. TGFβ1 and BMP7 are two central members in the TGFβ superfamily that each have different effects on EMT regulation. We recently reported that BMP7 does not modify TGFβ1-stimulated phosphorylation of the TGFβ receptor, but significantly inhibited activation of EMT-related genes by TGFβ1 in BC cells, thereby reducing TGFβ1-mediated cell growth and metastasis [16]. However, the regulation of BMP7 in BC remains unclear.

MicroRNAs (miRNAs) are non-coding small RNAs that regulate gene expression at a post-transcriptional level, through specific binding to the 3′-untranslated region (3′-UTR) of target mRNA [17–19]. Specifically, miRNAs have been shown to play an important role in the tumorigenesis of BC [20–28], and in the control of BMP7 activation [29–32]. However, previous studies on miR-137 never addressed BMP7 as a potential target [33–37].

Here, we studied the expression of BMP7 and miR-137 in BC tissues, and investigated the association of miR-137 levels with patient prognosis. We used bioinformatics analyses to elucidate the interaction between miR-137 and BMP7. We then overexpressed miR-137 or inhibited miR-137 in 2 established BC cell lines *in vitro*, and examined their effects on BMP7 activation and BC cell invasion.

## RESULTS

### Association of high BC miR-137 levels with poor patient prognosis

**Figure 1:**
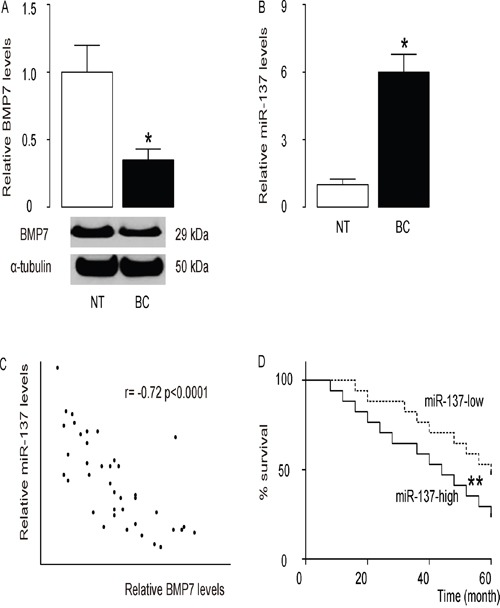
High miR-137 levels in BC specimens is associated with poor prognosis **A-C**. The levels of BMP7 and miR-137 in 40 pairs of BC tissues and adjacent non-tumor breast tissues (NT) were measured by Western blot (A) and RT-qPCR (B). **C**. A correlation test was performed between BMP7 and miR-137, using the 40 BC specimens. **D**. The 40 BC patients were followed-up for 60 months. The median value of all 40 cases was chosen as the cutoff point for separating miR-137-high cases (n=20) from miR-137-low cases (n=20). Kaplan-Meier curves were performed to compare 5-year survival between two groups. *p<0.05. **p<0.01. N=40.

The levels of BMP7 and miR-137 in 40 pairs of resected BC tissues (Stage IV) and adjacent non-tumor breast tissues (NT) were measured by Western blot and RT-qPCR, respectively (Table 1). BC specimens contained significantly lower levels of BMP7 (Figure 1A), and significantly higher levels of miR-137 (Figure 1B). We then performed a correlation test using these 40 BC specimens, and detected a strong inverse correlation between BMP7 and miR-137 (Figure 1C, ɤ=-0.72, p<0.0001, N=40), indicating a possible regulatory relationship between miR-137 and BMP7 in BC. These patients were followed up for 60 months to assess overall survival. The relationship of miR-137 or BMP7 levels and clinicopathological characteristics was evaluated using multivariate Cox regression analysis, showing that both were significantly associated with survival of the BC patients (Table 2). Next, the median value for miR-137 in these patients was used as the cutoff point for separating miR-137-high cases (n=20) from miR-137-low cases (n=20). Kaplan-Meier curves showed that patients with high miR-137 levels in BC tissue had a significantly lower 5-year survival than those with low miR-137 levels in BC tissue (Figure 1D). These data suggest that high miR-137 levels in BC specimens may be associated with reduced patient survival.

**Table 1:** Clinical-pathological characteristics (total)

	Patients (n; %)	p
BC tissue/ Normal tumor-adjacent tissue	40 (100%) /40 (100%)	
Age (<60/≥60 years old)	12 (30%) /28 (70%)	0.62
Gender (male/female)	0 (0%) /40 (100%)	
Tumor site (breast)	40 (100%)	
Tumor grade (well or moderate/poor)	0 (0%) /16 (40%) /24 (60%)	0.008
Tumor stage (I/II/III/IV)	0 (0%) /0 (0%) /20 (50%) /20 (50%)	0.005
Lymph node metastasis (no/yes)	0 (0%) /40 (100%)	0.003
Distal metastasis at diagnosis (no/yes)	40 (0%) /0 (0%)	0.003

**Table 2:** Analysis of the prognostic values of miR-137 and BMP7 in BC patients by Cox regression model

	HR	95% Cl	P value
miR-137 (high vs low)	5.11	3.13-9.92	0.005
BMP7 (low vs high)	4.42	2.21-7.97	0.004

### MiR-137 inhibits BMP7 protein translation in BC cells

**Figure 2:**
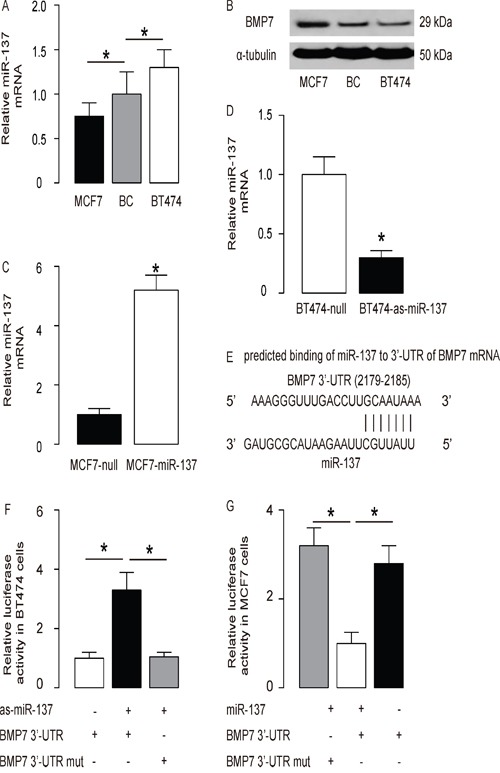
MiR-137 targets BMP7 to inhibit its protein translation in BC cells **A-B**. The levels of miR-137 by RT-qPCR (A) and BMP7 by Western blot (B) in BC cell lines BT474 and MCF7, compared to BC tissue from patients. **C**. MCF7 cells were transfected with miR-137 mimics (miR-137) or null as a control and examined for miR-137 levels. **D**. BT474 cells were transfected with antisense for miR-137 (as-miR-137) or null as a control and examined for miR-137 levels. **E**. Prediction of miR-137-binding sites on BMP7 mRNA by bioinformatics algorithms. **F-G**. The intact 3'-UTR of BMP7 mRNA (BMP7 3'-UTR), together with a 3'-UTR with mutant at miR-137-binding site of BMP7 mRNA (BMP7 3'-UTR mut), was then cloned into luciferase reporter plasmids. Luciferase activity was determined in BT474 cells, which were co-transfected with 1μg as-miR-137/null plasmids and 1μg BMP7 3'-UTR or BMP7 3'-UTR mut plasmids (F), and in MCF7 cells, which were co-transfected with 1μg miR-137/null plasmids and 1μg BMP7 3'-UTR or BMP7 3'-UTR mut plasmids (G). *p<0.05. N=5.

Next, we examined the levels of miR-137 and BMP7 levels in diffirent BC cell lines. We found that BT474 expressed relatively high level of miR-137 and relatively low level of BMP7, while MCF7 expressed relatively low level of miR-137 and relatively high level of BMP7 (Figure 2A-2B). Next, we transfected MCF7 cells with miR-137 (Figure 2C), and transfected BT474 cells with antisense for miR-137 (as-miR-137) (Figure 2D). The cells transfected with a null sequence were used as a control (null). The levels of miR-137 in these modified BC cells were assayed by RT-qPCR. The alterations of miR-137 levels in these cells were confirmed (Figure 2C-2D). These miR-137-modified BC cells were used to examine the functional binding of miR-137 to BMP7 mRNA predicted by bioinformatics algorithms (Figure 2E, Table 2). The intact 3'-UTR of BMP7 mRNA (BMP7 3'-UTR) and a 3'-UTR with mutant at miR-137-binding site of BMP7 mRNA (BMP7 3'-UTR mut) were prepared and then cloned into luciferase reporter plasmids. First, BT474 cells were co-transfected with 1μg as-miR-137/null plasmids and 1μg BMP7 3'-UTR or BMP7 3'-UTR mut plasmids (Figure 2F). Next, MCF7 cells were co-transfected with 1μg miR-137/null plasmids and 1μg BMP7 3'-UTR or BMP7 3'-UTR mut plasmids (Figure 2G). The results show that miR-137 specifically targets the 3’-UTR of BMP7 mRNA to inhibit its translation in BC cells.

### MiR-137 decreases BMP7 protein but not mRNA in BC cells

**Figure 3:**
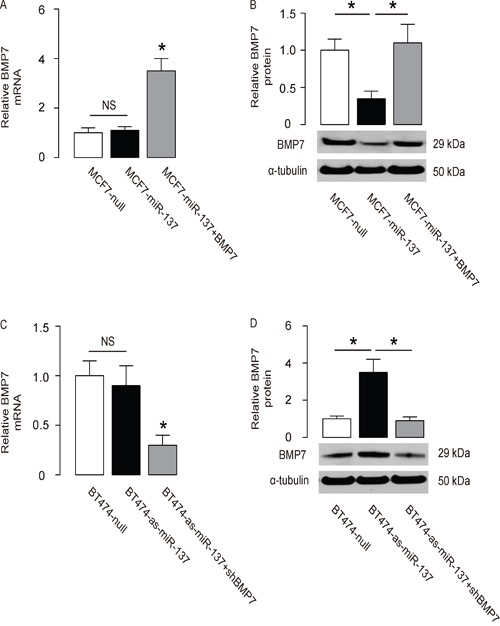
MiR-137 decreases BMP7 protein but not mRNA in BC cells **A-B**. The BMP7 levels in miR-137-overexpressing (and BMP7-overexpressing) MCF7 cells by RT-qPCR (A) and by Western blot (B). **C-D**. The BMP7 levels in miR-137-depleted (and BMP7-depleted) BT474 cells by RT-qPCR (C) and by Western blot (D). *p<0.05. NS: non-significant. N=5.

The effects of miR-137 on BMP7 were then examined in BC cells. The BMP7 mRNA did not alter (Figure 3A), but the BMP7 protein was significantly decreased in miR-137-overexpressing MCF7 cells (Figure 3B). Moreover, the BMP7 mRNA did not alter (Figure 3C), but the BMP7 protein was significantly increased in miR-137-depleted BT474 cells (Figure 3D).

### MiR-137 reduces BC cell EMT and invasion

**Figure 4:**
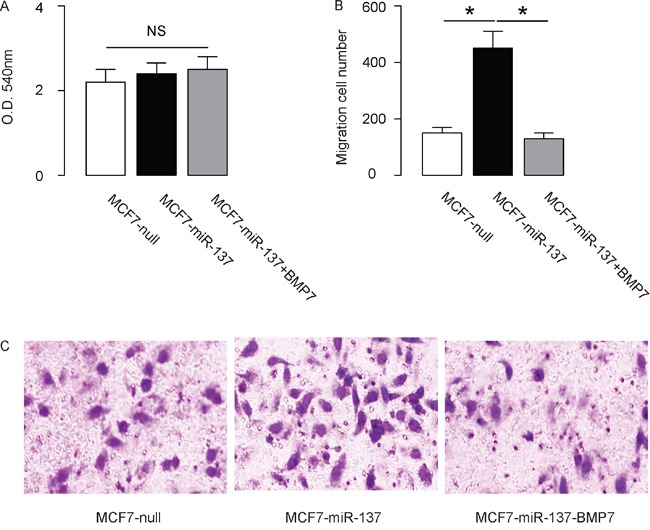
Overexpression of miR-137 increases MCF7 EMT and cell invasion through suppressing BMP7 **A-C**. MCF7 cell invasion by miR-137 overexpression (and BMP7 overexpression) in a transwell cell invasion assay, shown by quantification (A-B), and by representative images (C). *p<0.05. N=5.

**Figure 5:**
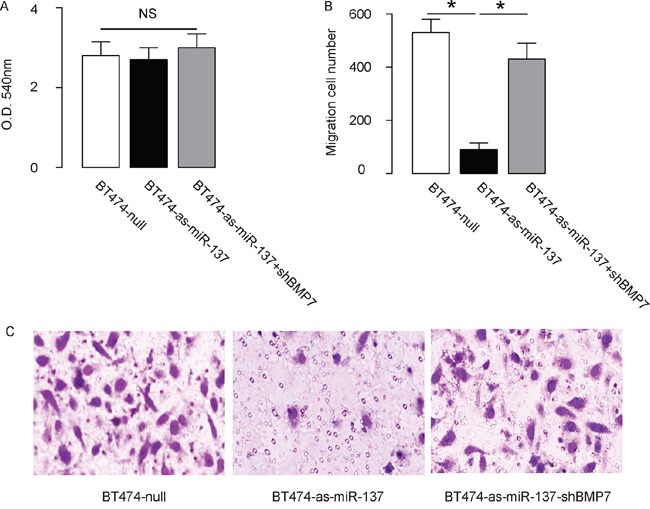
Depletion of miR-137 decreases BT474 EMT and cell invasion through augmentation of BMP7 **A-C**. BT474 cell invasion by miR-137 depletion (and BMP7 depletion) in a transwell cell invasion assay, shown by quantification (A-B), and by representative images (C). *p<0.05. N=5.

The effects of miR-137 modification on the EMT and invasion of cultured BC cells were then investigated. We found that miR-137 overexpression in MCF7 cells did not alter cell growth in an MTT assay (Figure 4A), but significantly increased the potential of EMT and cell invasion in a transwell cell migration assay (Figure 4B-4C). Moreover, miR-137 depletion in BT474 cells did not alter cell growth in an MTT assay (Figure 5A), but significantly decreased the potential of EMT and cell invasion in a transwell cell migration assay (Figure 5B-5C). Thus, MiR-137 decreases BC cell EMT and invasion.

### MiR-137 enhances BC cell invasion by suppressing BMP7

**Figure 6:**
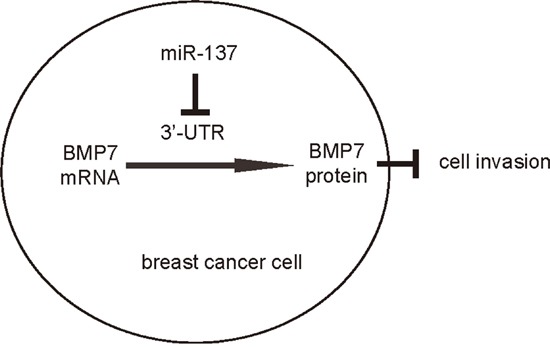
Schematic of the model MiR-137 enhances BC cell EMT and invasion, through translational suppression of BMP7.

In order to ascertain whether miR-137 promotes BC cell invasion through suppressing BMP7, we prepared plasmids for BMP7 overexpression (BMP7) and depletion (shBMP7). First, MCF7-miR-137 cells were further transfected with BMP7, which increased BMP7 mRNA (Figure 3A) and protein (Figure 3B) in these cells. Overexpression of BMP7 abrogated the promoting effects of miR-137 on the EMT and cell invasion in MCF7 cells (Figure 4B-4C), without affected cell growth (Figure 4A). Next, BT474-as-miR-137 was further transfected with shBMP7, resulting in decreases in BMP7 mRNA (Figure 3C) and protein (Figure 3D) in these cells. We found that the effects of as-miR-137 on BMP7 protein compromised the effects of shBMP7 on BMP7 protein, which explained the findings in BC cells transfected with both as-miR-137 and shBMP7. We found that BMP7 suppression abolished the inhibitory effects of as-miR-137 on EMT and cell invasion in BT474 cells (Figure 5B-5C), without affected cell growth (Figure 5A). Thus, miR-137 may enhance BC cell invasion by suppressing BMP7 (Figure 6).

## DISCUSSION

The inhibitory role of BMP7 in BC EMT and invasion has been well documented in the past studies. However, the regulation of BMP7 by miRNAs was only recently reported in lung cancer [42]. Yang et al. reported that miR-137 was significantly down-regulated in NSCLC tissues and cell lines. An *In vitro* functional assay demonstrated that over-expression of miR-137 inhibited lung cancer cell proliferation, migration and invasion, suggesting that miR-137 could act as a tumor suppressor in lung cancer progression. In addition, they identified BMP7 as a target of miR-137 in lung cancer cells, and used a luciferase reporter assay to show that miR-137 directly targeted 3'-UTR of BMP7. Furthermore, they showed that re-expression of BMP7 substantially reversed the tumor suppressive effects of miR-137 on lung cancer cell proliferation, migration, and invasion [42]. This study is interesting but also suggests that the role of a molecular could be very different from cancer to cancer, since BMP7 is believed to be a tumor suppressor in many cancers [43–46].

Here, we used bioinformatics analyses to screen all miRNAs that target BMP7, and we focused on the expression levels of those which were altered in BC specimens compared to normal tissue control. We found miR-137 to be one such microRNA. To the best of our knowledge, this follow-up study of our previous work [16] is the first study showing that BMP7 protein levels could be regulated by a miRNA in BC. High level of miR-137 in BC tissues was associated with poor prognosis in BC patients. We thus designed *in vitro* experiments to show a regulatory relationship between miR-137 and BMP7 in BC cells, which was consistence with the clinic findings showing an inverse correlation of these two factors in BC specimens.

In addition to regulation of BMP7 by miRNAs, BMP7 protein levels are modulated at the level of degradation, such as through protein ubiquitination. Moreover, miR-137 may have targets other than BMP7, and these targets should be analyzed to have an overview of the effects of miR-137 in the carcinogenesis of BC. Besides, future studies may also address the regulation of miR-137 in BC and confirm this model *in vivo*.

To summarize, the current study may provide evidence for using miR-137 as a specific target for future BC therapies.

## MATERIALS AND METHODS

### Experimental protocol approval

All experimental protocols were approved by the Research Bureau of Shanghai Jiao Tong University Affiliated Sixth People's Hospital. All mouse experiments were approved by the Institutional Animal Care and Use Committee at Shanghai Jiao Tong University Affiliated Sixth People's Hospital (Animal Welfare Assurance). Animal and human specimens were handled according to previously established guidelines.

### Patient specimens

Surgical BC resected specimens were obtained from 40 BC patients (all Stage III or IV) and paired adjacent non-tumor breast tissues (NT) in Shanghai Jiao Tong University Affiliated Sixth People's Hospital from 2008 to 2010 (Table 1). All patients were followed-up for 60 months, before which they obtained Informed consent and provided signed agreement about this study. The histology of the resected tissue were examined and determined independently by 2 senior pathologists.

### Culturing and transfection of BC cells

Human BC cell lines MCF7 [38] and BT474 [39] were originated from adenocarcinoma and ductal carcinoma, respectively. Both lines were purchased from ATCC (American Type Culture Collection, Manassas, VA, USA), and cultured in in RPMI1640 medium (Invitrogen, Carlsbad, CA, USA) supplemented with 15% fetal bovine serum (FBS; Sigma-Aldrich, St Louis, MO, USA) in a humidified chamber with 5% CO_2_ at 37 °C. All constructs were purchased from Origene (Beijing, China). Transfection was performed with 50nmol/l plasmids, using Lipofectamine 2000 (Invitrogen). The transfection efficiency (>95%) was determined based on expression of GFP in the transfected cells.

### Transwell cell invasion assay

Transwell cell invasion assay was performed as has been described previously [16].

### Cell growth assay

An MTT Kit (MTT, Roche, USA) was used for analyzing cell growth.

### MiRNA target prediction and 3'-UTR luciferase-reporter assay

MiRNAs targets were predicted using the algorithms from TargetScan [40]. The data were analyzed as previously described [41]. The candidate miRNAs were analyzed for context+ score (Supplementary Table 1). The BMP7 3'-UTR reporter plasmid (pRL-BMP7) and the BMP7 3'-UTR reporter plasmid with a mutant at miR-137 binding site (pRL-BMP7-mut) were both purchased from Creative Biogene (Shirley, NY, USA). Dual-luciferase reporter assay (Promega, Fitchburg, WI, USA) was performed according to the instructions from manufacturer.

### Quantitative RT-PCR (RT-qPCR)

Quantitative RT-PCR (RT-qPCR) was performed as has been described previously [16].

### Western blot

Western blot was performed as previously described [16].

### Statistical analysis

All statistical analyses were performed using the GraphPad Prism 6 (GraphPad Software, San Diego, CA, USA). Statistical analysis of group differences was carried out using a one-way analysis of variance (ANOVA) test followed by followed by Turkey multiple comparison post-hoc analysis. The relationship between miR-137 levels and clinicopathological characteristics was evaluated using multivariate Cox regression analysis. Bivariate correlations were calculated by Spearman's Rank Correlation Coefficients. Patients’ survival was determined by Kaplan-Meier analysis. All values represent the mean ± standard deviation (SD). A value of p<0.05 was considered statistically significant after Bonferroni correction.

## SUPPLEMENTARY MATERIALS TABLES


