# Supplementary material for: MicroRNA-137 inhibits BMP7 to enhance the epithelial-mesenchymal transition of breast cancer cells
Source: Oncotarget. 2017 Feb 17;8(11):18348–58. doi: 10.18632/oncotarget.15442 (PMC5392333; doi:10.18632/oncotarget.15442)
Supplement: Supplementary file 1 [file oncotarget-08-18348-s001.pdf]

MicroRNA-137 inhibits BMP7 to enhance the epithelial-mesenchymal transition of breast cancer cells

SUPPLEMENTARY TABLE

Supplementary Table 1: Prediction of human BMP7-binding miRNAs by Bioinformatics

miRNA	Position in the UTR	seed match	context++ score
miR-1298-5p	451-458	8mer	-0.36
miR-758-3p	439-445	7mer-m8	-0.3
miR-873-5p.1	424-430	7mer-m8	-0.17
miR-542-3p	438-444	7mer-1A	-0.17
miR-367-3p	2178-2184	7mer-1A	-0.16
miR-137	2179-2185	7mer-1A	-0.15
miR-363-3p	2178-2184	7mer-1A	-0.14
miR-25-3p	2178-2184	7mer-1A	-0.13
miR-1298-5p	456-462	7mer-1A	-0.12
miR-92a-3p	2178-2184	7mer-1A	-0.12
miR-92b-3p	2178-2184	7mer-1A	-0.12
miR-32-5p	2178-2184	7mer-1A	-0.11
miR-5688	2058-2064	7mer-m8	-0.1
miR-495-3p	2058-2064	7mer-m8	-0.09
miR-30c-5p	616-622	7mer-m8	-0.02
miR-30a-5p	616-622	7mer-m8	-0.02
miR-30d-5p	616-622	7mer-m8	-0.02
miR-30e-5p	616-622	7mer-m8	-0.02
miR-30b-5p	616-622	7mer-m8	-0.02

For each predicted target of each miRNA, the sum of the context+ scores for the sites to that miRNA was calculated as the total context+ score. Predicted targets of each miRNA family are sorted by total context+ score. The representative miRNA is the miRNA in its family with the most favorable (lowest) total context+ score. From the all candidates, only significant alteration of miR-137 was detected in human breast cancer specimens and paired non-tumor tissue.
